# Antigen Loading (e.g., Glutamic Acid Decarboxylase 65) of Tolerogenic DCs (tolDCs) Reduces Their Capacity to Prevent Diabetes in the Non-Obese Diabetes (NOD)-Severe Combined Immunodeficiency Model of Adoptive Cotransfer of Diabetes As Well As in NOD Mice

**DOI:** 10.3389/fimmu.2018.00290

**Published:** 2018-02-16

**Authors:** David P. Funda, Jaroslav Goliáš, Tomáš Hudcovic, Hana Kozáková, Radek Špíšek, Lenka Palová-Jelínková

**Affiliations:** ^1^Institute of Microbiology of the Czech Academy of Sciences, v.v.i., Prague, Czechia; ^2^Institute of Microbiology of the Czech Academy of Sciences, v.v.i., Nový Hrádek, Czechia; ^3^SOTIO a s., Prague, Czechia; ^4^Department of Immunology, 2nd Medical School, Charles University, Prague, Czechia

**Keywords:** type 1 diabetes, cell therapy, autoantigen, tolerogenic, dendritic cells, glutamic acid decarboxylase 65, non-obese diabetes-severe combined immunodeficiency mouse, non-obese diabetes mice

## Abstract

Tolerogenic DCs (tolDCs) are being researched as a promising intervention strategy also in autoimmune diseases including type 1 diabetes (T1D). T1D is a T-cell-mediated, organ-specific disease with several well-defined and rather specific autoantigens, i.e., proinsulin, insulin, glutamic acid decarboxylase 65 (GAD65), that have been used in animal as well as human intervention trials in attempts to achieve a more efficient, specific immunotherapy. In this study, we have tested tolerogenic DCs for their effectiveness to prevent adoptive transfer of diabetes by diabetogenic splenocytes into non-obese diabetes (NOD)-severe combined immunodeficiency (NOD-SCID) recipients. While i.p. application of tolDCs prepared from bone marrow of prediabetic NOD mice by vitamin D2 and dexamethasone significantly reduced diabetes transfer into the NOD-SCID females, this effect was completely abolished when tolDCs were loaded with the mouse recombinant GAD65, but also with a control protein—ovalbumin (OVA). The effect was not dependent on the presence of serum in the tolDC culture. Similar results were observed in NOD mice. Removal of possible bystander antigen-presenting cells within the diabetogenic splenocytes by negative magnetic sorting of T cells did not alter this surprising effect. Tolerogenic DCs loaded with an immunodominant mouse GAD65 peptide also displayed diminished diabetes-preventive effect. Tolerogenic DCs were characterized by surface maturation markers (CD40, CD80, CD86, MHC II) and the lipopolysaccharide stability test. Data from alloreactive T cell proliferation and cytokine induction assays (IFN-γ) did not reveal the differences observed in the diabetes incidence. Migration of tolDCs, tolDCs-GAD65 and tolDCs-OVA to spleen, mesenteric- and pancreatic lymph nodes displayed similar, mucosal pattern with highest accumulation in pancreatic lymph nodes present up to 9 days after the i.p. application. These data document that mechanisms by which tolDCs operate *in vivo* require much better understanding for improving efficacy of this promising cell therapy, especially in the presence of an antigen, e.g., GAD65.

## Introduction

Type 1 diabetes (T1D) is a T cell-mediated disease, in which both CD4 and CD8 T cells are necessary and sufficient in precipitating the disease by targeting very specifically relatively small volume of highly specialized beta-cells within Islets of Langerhans (1, 2). Non-obese diabetes (NOD) mice and NOD-severe combined immunodeficiency (NOD-SCID) mice represent well studied and frequently used animal models of T1D. While the NOD spontaneous mouse model allows to study the natural course of the disease in its complexity, NOD-SCID mice with adoptively cotransferred diabetogenic NOD splenocytes are often used for testing regulatory/protective capacity of various cell subsets in a shorter timeframe (3).

Dendritic cells (DCs) are highly effective specialized antigen-presenting cells (APCs) and central regulators of immune responses—they are important for induction of effector immune responses, but depending on their developmental stage or environment/culture conditions, also promote tolerance by various mechanisms: T-cell anergy, T-cell deletion, induction of different subsets of Tregs, such as CD8^+^ Tregs, Foxp3^+^ Tregs, or Tr1 cells (4), or even induction of a Th2 shift (5) or Bregs (6).

The first animal study dealing with DCs in T1D prevention showed that DCs isolated from pancreatic and not other lymph nodes lowered diabetes incidence when reinjected to 4-week-old NOD mice (7). It has been shown that bone marrow-derived tolerogenic DCs (tolDCs) generated in the presence of GM-CSF and IL-4 in an antigen-nonspecific manner displayed diabetes-preventive properties (8, 9). Antigen-nonspecific treatment with a pegylated TLR7 ligand *in vivo* induced tolDCs and decreased diabetes in NOD mice (10). Administration of DCs prepared in the presence of interleukin 10 (IL-10) with (11) or without (12) antigen supply both prevented diabetes and insulitis in NOD mice. In addition, tolDCs pulsed with apoptotic bodies containing beta-cell antigens decreased diabetes and insulitis in a transgenic NOD model of accelerated diabetes (13). While data from Pujol-Autonell et al. documented that reverting diabetes in already diabetic animals might be difficult (14), genetically engineered bone marrow-derived DCs transduced with IL-4 were able to prevent diabetes in 12-week-old prediabetic NOD mice with advanced insulitis (15).

Thus, tolDCs represent a promising strategy in T1D prevention at high-risk individuals or even treatment of the disease. The first human phase I trial of autologous tolDCs in T1D was completed (16, 17) and another, based on proinsulin-loaded tolDCs, has been opened (18).

Apart from the efficacy of tolDCs to suppress the disease in animal models, preferably also at later stages before or even after clinical onset of T1D, several other important parameters must be taken into account, such as their stability, survival, expression of costimulatory and homing molecules, migration, dying pathway, antigen-specificity or requirement, and optimal application route (4, 19). We have been involved in testing and optimizing tolDC protocol based on GM-CSF and IL-4 cell culture with added dexamethasone and vitamin D2 followed by activation of tolDCs by lipopolysaccharide (LPS) analog monophosphoryl lipid A (MPLA). This protocol was developed according to the good manufacturing practice standards for preparation of human tolDCs that are stable under inflammatory conditions (20). Indeed, it would be desirable to make this protocol antigen-specific by using safely a beta-cell specific antigen for targeting the pathological immune reaction more effectively, as it has been researched in experimental autoimmune encephalomyelitis (EAE) (21, 22) or experimental arthritis (23, 24), but less clear-cut in case of T1D (8, 9, 11, 13).

Thus, the initial aim of this study was to test this human tolDC protocol in NOD-SCID mice in an antigen-specific manner by using mouse recombinant glutamic acid decarboxylase 65 (GAD65) naturally processed by tolDCs. Surprisingly, GAD65-loaded tolDCs (tolDCs-GAD65) while keeping their surface characteristics as well as their allogeneic proliferative and cytokine induction properties lost their diabetes-preventive effect. Diabetes incidence was also assessed in the NOD mouse model. Some possible mechanisms, other antigens, culture conditions as well as migration patterns are addressed or excluded in this study.

## Materials and Methods

The minimum information about tolerogenic antigen-presenting cells (MITAP) checklist was followed for the preparation of this manuscript (25).

### Animals

Female NOD, NOD-SCID, and C57BL/6 mice were purchased from Taconic (Albany, NY, USA) whereas female C57BL/6 mice were obtained from the animal facility of the Institute of Physiology, Czech Acad. Sci., Prague, Czech Republic and used in experiments as described below at 6–13 weeks of age. The mice were maintained in the specific pathogen-free animal facilities under standard light- and climate-controlled conditions, fed standard Altromin 1414 diet, and water was provided *ad libitum*. All experiments were approved by our institutional animal ethics office (Laboratory Animal Care and Use Committee of the Institute of Microbiology v.v.i., Academy of Sciences of the Czech Republic, approval ID: 94/2006 and 244/2009) in strict accordance with the Federation of European Laboratory Animal Science Associations guidelines. Endpoint criteria were established to minimize suffering and ensure animal welfare.

### DC Generation

Mouse bone marrow dendritic cells were generated from femur and tibia of 8- to 10-week-old female NOD mice, which were surgically removed postmortem. The bone marrow was flushed with a syringe/needle combination. Erythrocytes were lysed using red blood cell lysing buffer (Sigma-Aldrich, St. Louis, MO, USA), isolated cells were washed and counted for absolute live cells quantity but without documenting their morphology. The fresh isolated cells were subsequently cultured (37°C, 5% CO_2_) in Petri cell-culture dishes (90 mm in diameter) in complete prewarmed l-glutamine containing RPMI-1640 (Lonza, Verviers, Belgium) supplemented with 10% heat-inactivated fetal bovine serum (FBS; Gibco-Life Technologies, Paisley, UK), 100× diluted NEM-Non-essential amino acid (Sigma-Aldrich), 1 µM sodium pyruvate (Sigma-Aldrich), 50 IU/mL penicillin, 50 µg/mL streptomycin, and 50 µM 2-β-mercaptoethanol. In other experiments, cells were cultured in serum-free (SF) CellGro medium (CellGenix, Freiburg, Germany) with the same supplements as for RPMI-1640 except FBS. Cells were plated at a density of 4 × 10^6^ cells/10 mL in the corresponding medium in the presence of GM-CSF (20 ng/mL) and IL-4 (4.5 ng/mL; PeproTech, Rocky Hill, NJ, USA) for 6 days. Fresh medium (10 mL) was added on day 3. At day 6, half (10 mL) of the medium was harvested, collected cells were counted, and resuspended in 10 mL of fresh medium and added back into the culture. Thus half of the medium was replaced with fresh one. Tolerogenic DCs were induced by adding dexamethasone (1 µM; Medochemie, Limassol, Cyprus) and vitamin D2 (1.5 ng/mL; Zemplar, AbbVie, North Chicago, IL, USA) on day 6, whereas immature DCs (iDCs) and control matured DCs [control matured bone marrow-derived dendritic cell (cDCs)] were generated without these tolerogenic factors. For antigen loading of tolDCs the mouse recombinant GAD65 was obtained from Sino Biological, Beijing, China, whereas Ovalbumin EndoGrade (OVA) was purchased from Hyglos, Regensburg, Germany and immunodominant peptide no. 35 of GAD65 sequence (purity 97%) from ThinkPeptides, Oxford, UK. At day 7, nonadherent cells were collected, washed, counted, and plated at a density of 1 × 10^6^ cells/mL in fresh medium on a 6-well plate (4 × 10^6^ cells per well). TolDCs were left unpulsed or loaded with GAD65 (tolDC-GAD65, 2 or 1 µg/mL), OVA (tolDC-OVA, 1 µg/mL), or GAD65-immunodominant peptide no. 35 (tolDC-pept, 1 µg/mL). After 4 h, all types of tolDCs as well as cDCs were finally activated with 2 µg/mL VacciGrade MPLA from S. minnesota R595 (MPLA; InvivoGen, Toulouse, France) for 22 h. At the end of the cell cultivation, the non-adherent cells were collected, counted for absolute live cells, and in the same culture medium immediately processed for follow-up experiments.

### Adoptive Transfer and Diabetes Monitoring

7- to 8-week-old NOD-SCID females were used as recipients in adoptive cotransfer experiments. Diabetogenic splenocytes were isolated from 12- or 13-week-old prediabetic NOD female mice. At day 8 of a cell culture, 3 × 10^6^ of live tolDCs, tolDCs-GAD65, tolDCs-OVA, or tolDCs-pept were resuspended in Phosphate Bovine Saline (PBS, Lonza) together with 5 × 10^6^ live diabetogenic splenocytes (erythrocytes were lysed with red blood cell lysing buffer and cells washed twice in PBS) and injected i.p. (left side of the belly) in a final volume of 300 µL of PBS. The Control group was injected with 5 × 10^6^ diabetogenic splenocytes in PBS. In another experiment, T cell-enriched splenocytes were prepared by a negative selection using EasySep Mouse T cell Enrichment Kit (Stemcell Technologies, Vancouver, BC, Canada). The T cell-enriched fraction had purity >92%. Equivalent of 33% of 5 × 10^6^ whole splenocytes, i.e., 1.65 × 10^6^ T cells were mixed with 3 × 10^6^ tolDCs or tolDCs-GAD65, and injected i.p. in a final volume 300 µL of PBS to NOD-SCID mice. The same procedure was used for adoptive cotransfer of tolDCs generated in SF CellGro medium. All recipient NOD-SCID mice were monitored for diabetes incidence weekly for min of 12 weeks. Tolerogenic DCs as well as tolDCs-GAD65 and tolDCs-OVA were also tested in the spontaneous NOD mouse model by a single dose of 3 × 10^6^ of live cells injected i.p. at age of 4 weeks. NOD mice were monitored for diabetes incidence from 8 weeks until age of 310 days. The diabetes onset was monitored once weekly from tail vein blood with the glucometer Freestyle Lite (Abbott Diabetes Care Ltd., Witney, UK) and diagnosis of diabetes was based on two consecutive blood glucose readings >12 mM in 3 days. The first reading was then used as the date of diabetes onset. Neither a NOD-SCID nor a NOD mouse displayed transient glycemia over 12 mM in this study. The glycemia measurement values for individual NOD mice are provided in the Table S1 in Supplementary Material.

### Flow Cytometry

Cells were stained with the following fluorochome-conjugated monoclonal antibodies: anti-CD3 (145-2C11), CD4 (GK1.5), CD8a (53-6.7), CD11c (N418), CD40 (1C10), CD80 (16-10A1), CD86 (GL1), MHC class II (I-A/I-E) (MS/114.15.2), CD103 (2E7), c-kit (ACK2), IL7Rα (A7R34), CCR5 (HM-CCR5), CCR7 (3D12), IFN-γ (XMG1.2), AnnexinV, and Fc block CD16/CD32 (Thermo Fisher Scientific, Waltham, MA, USA). Cells were incubated in PBS containing 2% FBS when stained for surface markers. Propidium iodide or Hoechst33342 were used for exclusion of dead cells or to assess the proportion of dead cells. HEPES buffer was used for AnnexinV staining. Cells were washed three times, then stained and kept in the HEPES buffer for flow cytometry analysis. For intracellular detection of IFN-γ on day 5 of allogeneic coculture, cells were restimulated *in vitro* with 25 ng/mL of phorbo-12-myristate-13-acetate and ionomycin (1 µg/mL, Sigma-Aldrich) for 4 h in the presence of monensin (2 µM, Thermo Fisher Scientific). Cells were first stained for surface markers then fixed/permeabilized with the Cytofix/Cytoperm kit (Thermo Fisher Scientific) following the manufacturer’s instruction. Unstimulated cells cultured in the presence of monensin were used as controls. Isotype control antibodies were included to determine the amount of nonspecific binding. Data were acquired by LSR II flow cytometer (BD Bioscience, San Diego, CA, USA) and analyzed using FlowJo software (Tree Star, Ashland, OR, USA).

### Stability Test and Cytokine Production

Stability test was carried out by stimulation of iDC, cDC, and all derived types of tolDCs with 1 µg/mL LPS from *Escherichia coli* 0111:B4 (LPS; Sigma-Aldrich) for additional 24 h. Cells were seeded in 96-well U-bottom plates at density 2 × 10^5^/200 μL. Unstimulated cells were used as controls. Expression of surface maturation markers (CD40, CD80, CD86, MHC II) was measured on live cells by flow cytometry. IL-10 and IL-12p70 were measured in tissue culture supernatants after MPLA activation or after the stability test (24 h LPS at 1 µg/mL) by ELISA DuoSet kits (R&D Systems, Minneapolis, MN, USA) according to the manufacturer’s instructions.

### Allogeneic DC/T Cell Cultures

To assess alloproliferative responses splenocytes isolated from C57BL/6 female mice were labeled for 5 min with 3 µM 5,6-carboxylfluorescein diacetate succinimidyl ester (CFSE; Thermo Fisher Scientific) and according to the manufacturer’s instructions. Labeled splenocytes were seeded in 96-well U-bottom plates at a concentration 2 × 10^5^/200 μL and cocultured in complete RPMI-1640 medium with 2 × 10^4^/200 μL (10:1 ratio) iDCs, cDCs, or different types of tolDCs for 3 and 5 days at 37°C and 5% CO_2_. CFSE dilution in live CD3^+^ T cells was assessed by flow cytometry. Unstained splenocytes cocultured with DCs were used in the same setting for allogeneic induction of IFN-γ after 5 days. The expression of IFN-γ was assessed by intracellular staining in CD3^+^CD4^+^ cells and flow cytometry.

### Autologous CD8^+^ T Cell-Mediated *In Vitro* Killing of tolDCs

Splenocytes were isolated from NOD females and CD4^+^ or CD8^+^ were isolated by negative magnetic selection using EasySep Mouse CD4^+^ and CD8^+^ T Cell Isolation Kit (Stemcell Technologies) according manufacturer’s instructions. Dendritic cells (iDCs, cDCs, tolDCs, tolDCs-GAD65, and tolDCs-OVA) were cocultured with autologous CD4^+^ or CD8^+^ splenic T cells at a 1:1 ratio and at a concentration 1 × 10^6^/mL in complete RPMI-1640 medium for 4, 8, 12, and 24 h. DCs without T cells were cultured as controls. In another experiment dendritic cells and autologous splenocytes were cultured in the same ratio (3:5) as used for i.p. administrations to NOD-SCID mice. Cell death was measured by AnnexinV and Hoechst33342 staining on CD3^-^CD11c^+^ cells by flow cytometry.

### DC Migration

Unloaded tolDCs and tolDCs-GAD65 were generated from 8-week-old-NOD females. The PKH26 Red Fluorescent Cell Linker kit (Sigma-Aldrich) was used for labeling DCs for *in vivo* migration experiment according to the manufacturer’s protocol. Briefly, cells were washed in RPMI-1640 medium without FBS, resuspended in Diluent Solution C (1 mL of diluent C/1 × 10^7^ cells), and stained with PKH26 for 5 min in room temperature with periodic mixing. The staining reaction was stopped by addition of FBS-supplemented RPMI-1640 medium and in which cells were then washed three times. Labeled tolDCs and tolDCs-GAD65 were applied i.p. (left side of the belly) to 6-week-old NOD female mice at dose of 5 × 10^6^ cells. The control group was injected with unlabeled tolDCs. Cell suspensions from spleen, mesenteric lymph nodes (MLNs), pancreatic lymph nodes (PLNs), and systemic inguinal lymph nodes (ILNs) were prepared after 3, 5, 7, 9, and 12 days and live PKH26^+^CD11^+^ were detected by flow cytometry.

### Statistical Analysis

All data are expressed as the mean ± SEM values. Statistical analyses were performed using GraphPad Prism 4 (GraphPad Software, La Jolla, CA, USA). The unpaired *t*-test and one-way ANOVA followed by Tukey’s multiple comparison posttest were used for evaluation of data from multiple measurements from two or multiple groups. Differences were considered statistically significant when *p*-value was <0.05. The cumulative diabetes incidence was assessed using the Kaplan–Meier estimation and contingency tables. Log-rank test and Chi-square test were used for comparisons between. The *p*-values were compensated for multiple comparison (Bonferroni) of survival curves for each experiment.

## Results

### Tolerogenic DCs but not tolDCs-GAD65 Prevent Diabetes in the Adoptive Transfer Model of NOD-SCID Mice

**Figure 1:**
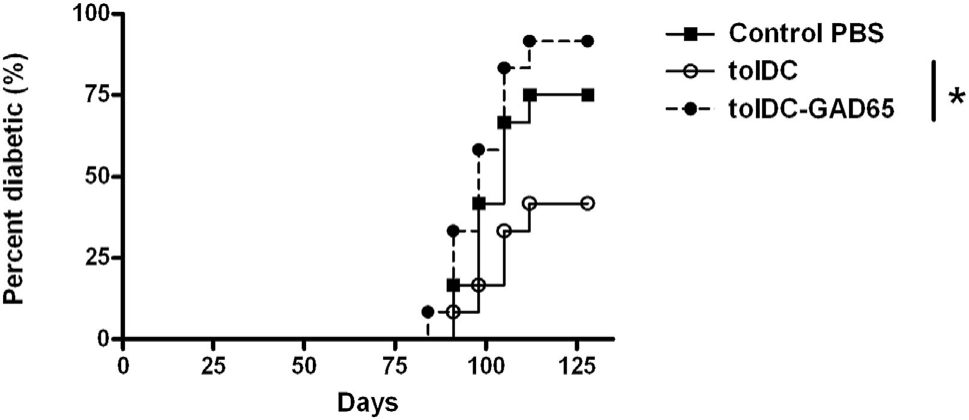
Tolerogenic DCs but not tolDCs-GAD65 prevent diabetes in the adoptive transfer model of non-obese diabetes (NOD)-severe combined immunodeficiency (NOD-SCID) mice. Tolerogenic DCs (tolDCs) or glutamic acid decarboxylase 65 (GAD65) (2 µg/mL) loaded tolDCs (tolDCs-GAD65) were generated from bone marrows of 8- to 10-week-old NOD females by cultivation in the presence of GM-CSF and IL-4 followed by additions of dexamethasone/vitamin D2 and monophosphoryl lipid A. Dendritic cells (3 × 10^6^) were resuspended in phosphate bovine saline (PBS) together with 5 × 10^6^ diabetogenic splenocytes from 13-week-old prediabetic NOD females (*n* = 8). Cells were then injected i.p. (left side of the belly) in a volume of 300 µL PBS to 8-week-old NOD-SCID female recipients (*n* = 12). Diabetogenic splenocytes in PBS were used as the Control group. Data are presented as cumulative diabetes incidence in NOD-SCID recipients, *p*-values were compensated for multiple comparisons, tolDC vs. tolDC-GAD65: *p = 0.0159.

In this initial experiment we tested whether i.p. administration of autoantigen-loaded tolDCs could increase the efficacy of the diabetes prevention by tolDCs in the NOD-SCID model of adoptive transfer of diabetes. Surprisingly, we found out that tolDCs that were cultured from day 7 with mouse GAD65 (2 µg/mL) completely lost their diabetes-preventive properties when cotransferred with diabetogenic splenocytes from 13-week-old prediabetic NOD females to NOD-SCID recipients (*n* = 12). As shown in Figure 1, i.p. application of 3 × 10^6^ unloaded tolDCs together with 5 × 10^6^ diabetogenic splenocytes led to substantial, although not statistically significant, reduction (75% to 42%) of diabetes onset in NOD-SCID recipients compared to the Control group injected with only diabetogenic splenocytes. In contrast, i.p. administration of tolDC exposed to mouse GAD65 (tolDC-GAD65) not only did not reduce diabetes incidence but, on the contrary, led to a more rapid onset and higher diabetes incidence than in the Control group receiving only diabetogenic splenocytes (Figure 1). Thus, the diabetes incidence in tolDC-GAD65 group was significantly higher than in group cotransferred with unloaded tolDCs (*p* = 0.0159). There was no difference in diabetes incidence between the tolDC-GAD65 and Control groups (*p* = 0.6651) (Figure 1).

The tolDCs and tolDCs-GAD65 were prepared by a standard 8-day protocol using RPMI-1640 medium supplemented with 10% FBS. Dexamethasone and vitamin D2 were added on day 6, mouse GAD65 on day 7. Dendritic cells were activated for the last 22 h by 2 µg/mL of VacciGrade MPLA. This unexpected result led us to repeat this initial experiment and carry out several modifications in order to clarify this phenomenon.

### Both GAD65 and OVA Abrogate Diabetes-Preventive Properties of tolDCs and This Effect Does Not Seem to Be due to the Presence of APCs within Diabetogenic Splenocytes

**Figure 2:**
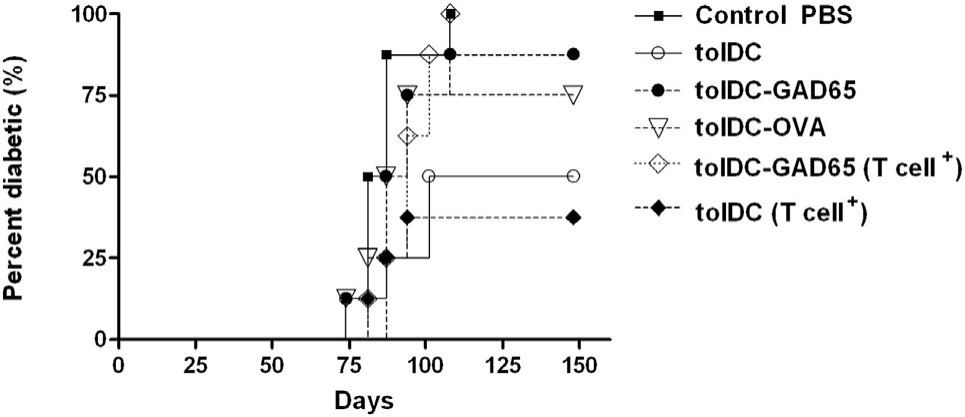
Antigen-loaded tolDCs fail to lower induction of diabetes in non-obese diabetes (NOD)-severe combined immunodeficiency (NOD-SCID) mice by transfer of diabetogenic NOD splenocytes but also by transfer of splenic T cells. Diabetogenic splenocytes (5 × 10^6^ per mouse) were isolated from 13-week-old prediabetic NOD females (*n* = 11). T cells were enriched (cell purity >92%) by negative selection (EasySep T cell Enrichment kit, Stemcell Tech.), and equivalent of 33% of splenocytes, i.e., 1.65 × 10^6^ T cells per mouse were used for diabetes induction in NOD-SCID recipients. Tolerogenic DCs, glutamic acid decarboxylase 65 (GAD65)- (1 µg/mL) or OVA- (1 µg/mL) loaded tolDCs were generated from bone marrows of 8- to 10-week-old NOD females by cultivation in the presence of GM-CSF and IL-4 followed by additions of dexamethasone/vitamin D2 and stabilized by monophosphoryl lipid A (MPLA). Diabetogenic splenocytes or enriched T cells (groups marked as T cell^+^) were resuspended in phosphate bovine saline (PBS) together 3 × 10^6^ tolDCs and injected i.p. (left side of the belly) in a volume of 300 µL PBS to 8-week-old NOD-SCID female recipients (*n* = 8). Diabetogenic splenocytes in PBS were used as the Control group. Data are presented as cumulative diabetes incidence in NOD-SCID recipients, *p*-values were compensated for multiple comparisons.

To confirm and extend the data from Figure 1, we have repeated these groups in a second adoptive cotransfer experiment in NOD-SCID mice. In order to clarify whether the effect is autoantigen (i.e., GAD65) specific we have also included a group of tolDCs loaded with a naturally processed control protein—OVA (tolDC-OVA). These tolDCs were again prepared in serum-supplemented RPMI-1640 medium, but this time loaded with a lower dose (1 µg/ml) of GAD65 or OVA on day 7, and activated by 22-h culture with 2 µg/ml of VacciGrade MPLA. Similar to previous experiment, unloaded tolDCs again markedly lowered diabetes incidence in NOD-SCID mice compared to the Control group injected with diabetogenic splenocytes alone (Figure 2), but the difference was not statistically significant due to multiple comparisons of six groups (*n* = 8). Tolerogenic DCs pulsed with GAD65, but also OVA, both failed to substantially lower the 100% diabetes transfer found in the Control group. The tolDC–OVA group displayed slightly lower diabetes incidence than the tolDC-GAD65 group, but this disease prevention was not at all statistically significant when compared to the Control group (Figure 2).

Next, we wanted to test whether other APCs present within the preparation of whole diabetogenic NOD splenocytes may be responsible for an aberrant immunogenic presentation of previously processed antigen (GAD65) by tolDCs. Thus, instead of whole splenocytes, we have used T-cell fraction, prepared by a negative T-cell enrichment (Stemcell Tech.) with T-cell purity over 92%. Approximately equivalent dose of T cells (1.65 × 10^6^) as present within the 5 × 10^6^ splenocytes was used for disease induction in the NOD-SCID recipients (T cell^+^ groups).

However, by using T cell-enriched splenocytes (T cell^+^) we observed again the same diabetes-preventive pattern of tolDCs and tolDCs-GAD65. Unloaded tolDCs substantially lowered the effect of diabetogenic T cells (100–50%) and slightly more compared to the whole diabetogenic splenocytes (100–37.5%), whereas autoantigen-loaded tolDCs-GAD65 again failed to prevent diabetes in NOD-SCID recipients. In fact, the course of the disease induction was closest between the Control and tolDC-GAD65 (T cell^+^) groups (Figure 2).

These data document, that APCs within diabetogenic splenocytes probably play an unimportant role in abolishing the disease preventive effect when mixed with tolDCs that naturally processed an autoantigen (GAD65). This experiment also showed that the decrease of diabetes-preventive potential of tolDCs does not seem to be GAD65-specific. Although OVA-loaded tolDCs prevented diabetes onset in two of eight mice, this reduction does not represent a substantial disease protection compared to the Control group (Figure 2).

### GAD65-Peptide Loaded tolDCs Failed to Prevent Diabetes in NOD-SCID Recipients and Serum-Free Conditions Do Not Alter the Effect of Antigen-Loaded tolDCs

**Figure 3:**
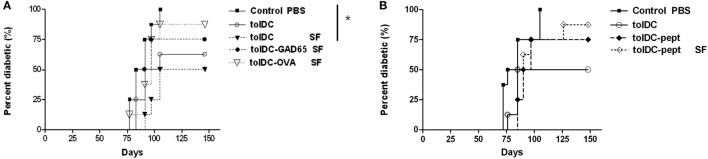
Serum-free cultured tolDCs and GAD65-loaded tolDCs in diabetes prevention using the non-obese diabetes (NOD)-severe combined immunodeficiency (NOD-SCID) model of adoptive transfer of diabetes. Dendritic cells were generated from bone marrows of 8- to 10-week-old NOD females by cultivation in the presence of GM-CSF and IL-4 followed by additions of dexamethasone/vitamin D2 and final maturation with monophosphoryl lipid A (MPLA). **(A)** Tolerogenic DCs loaded with 1 µg/mL of glutamic acid decarboxylase 65 (GAD65) (tolDC-GAD65) or OVA (tolDC-OVA) were prepared in SF medium, whereas tolDCs were cultured in both serum-supplemented (10% fetal bovine serum RPMI-1640) and SF media (tolDC SF). **(B)** In another experiment, unloaded tolDCs were compared to tolDCs loaded with 1 µg/mL of the GAD65-immunodominant peptide no. 35 (tolDC-pept) and prepared in both serum-supplemented and SF media. Diabetogenic splenocytes (5 × 10^6^ per mouse) from 12-week-old prediabetic NOD females (*n* = 10) and above listed groups of tolDCs (3 × 10^6^) were mixed and applied i.p. (left side of the belly) in a volume of 300 µL phosphate bovine saline (PBS) to 7-week-old NOD-SCID female recipients (*n* = 8). Diabetogenic splenocytes in PBS were used as the Control group in both experiments. Data are presented as cumulative diabetes incidence in NOD-SCID recipients and *p*- values were compensated for multiple comparisons **(A)** Control PBS vs. tolDC SF: *p = 0.026.

In order to determine in the literature (26) addressed effect of FBS on antigen-specific tolerance induction by tolDCs as well as to reflect the fact that DCs for human trials are prepared in SF conditions, we have compared the diabetes-preventive capacity of unloaded tolDCs cultured in serum-supplemented vs. SF conditions as well as retested effects of tolDCs-GAD65 and tolDCs-OVA in SF conditions (Figure 3A). Compared to the Control group of NOD-SCID mice (*n* = 8) administered with only 5 × 10^6^ of diabetogenic splenocytes, antigen-unloaded tolDCs again markedly reduced diabetes incidence in serum-supplemented conditions (100–50% in both Figures 3A,B). The difference was even more remarkable (100–50%) due to the course of the diabetes onset and thus statistically significant in SF conditions (*p* = 0.026) (Figure 3A).

On the other hand, antigen-loaded tolDCs-GAD65 and tolDCs-OVA prepared in SF conditions did not substantially decrease diabetes incidence in the NOD-SCID recipients, but this time tolDC-GAD65 and tolDC-OVA groups (Figure 3A) displayed a reversed pattern as in Figure 2. Thus, the presence of FBS did not alter disease-preventive properties of tolDCs. Last but not least, we have also tested not a whole protein, but the GAD65-immunodominant peptide no. 35 (pept) that is identical in murine and human GAD65 (27). As presented in Figure 3B, this immunodominant peptide added to tolDCs at concentration of 1 µg/mL and naturally processed by DCs (tolDC-pept) also failed to statistically significantly prevent diabetes in the NOD-SCID recipients in both SF and serum-supplemented conditions. Thus, in all three experiments only antigen-unloaded tolDCs exhibited substantial effects on disease prevention using the NOD-SCID mouse model of adoptive cotransfer of diabetes.

### Diabetes- Preventive Effect of tolDCs and Antigen-Loaded tolDCs in NOD Mice

**Figure 4:**
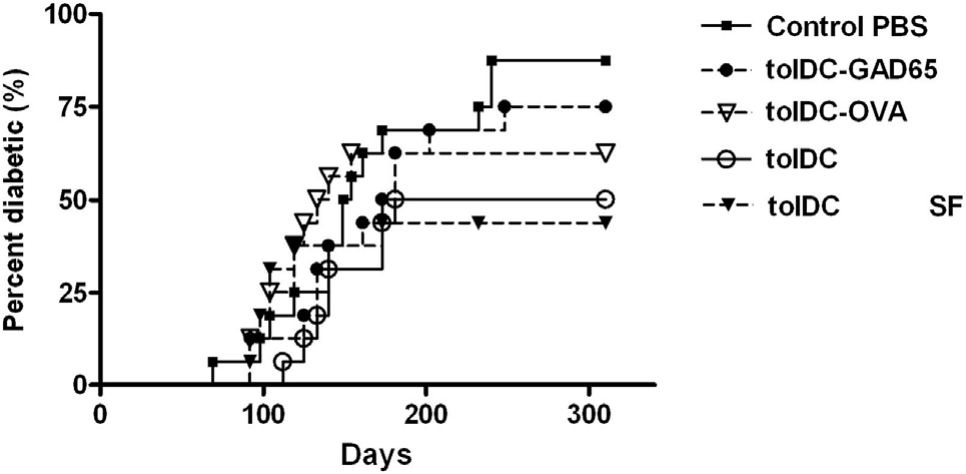
Diabetes-preventive effect of tolDCs but not antigen-loaded tolDCs in the spontaneous model of type 1 diabetes—the non-obese diabetes (NOD) mice. Tolerogenic DCs, GAD65- (1 µg/mL) or OVA- (1 µg/mL) loaded tolDCs were generated from bone marrows of 8- to 10-week-old NOD females by cultivation in serum-supplemented (10% fetal bovine serum RPMI-1640) medium in the presence of GM-CSF and IL-4 followed by additions of dexamethasone/vitamin D2 and stabilized by MPLA. Tolerogenic DCs were also prepared in SF media (tolDC SF). TolDCs (3 × 10^6^) were resuspended in phosphate bovine saline (PBS) and injected i.p. (left side of the belly) in a volume of 200 µL to 4-week-old NOD females (*n* = 16). i.p. application of 200 µL PBS alone was used for the Control group. Diabetes incidence was observed weekly (from week 12) until the age of 310 days. Data are presented as cumulative diabetes incidence, *p*-values were compensated for multiple comparisons.

To further assess the effect of antigen loading and serum-free conditions on the diabetes-preventive properties of tolDCs, selected groups of tolDCs were tested also in the spontaneous NOD mouse model of T1D. While control animals displayed diabetes incidence of 87.5% at age of 310 days, the group injected with a single dose of 3 × 10^6^ unloaded tolDCs showed substantially reduced diabetes incidence to 50%, *n* = 16 (Figure 4). Similar effect was observed when using unloaded tolDCs prepared in serum-free conditions, that lowered diabetes incidence to 43.8%, but this group displayed a faster initial onset of diabetes. On the other hand, antigen-loaded tolDC-GAD65 and tolDC-OVA prepared in serum-supplemented conditions did not substantially decreased diabetes incidence compared to the Control group (75 and 62.5% diabetic animals, respectively) (Figure 4). Thus, although the comparison of the multiple groups with the Control group was not statistically significant (*p* values corrected for multiple comparisons), data obtained in NOD mice paralleled those from the NOD-SCID model of adoptive cotransfer of diabetes (Figures 1, 2 and 3A,B).

### No Substantial Differences in Phenotype and Stability of tolDCs and Antigen-Loaded tolDCs

**Figure 5:**
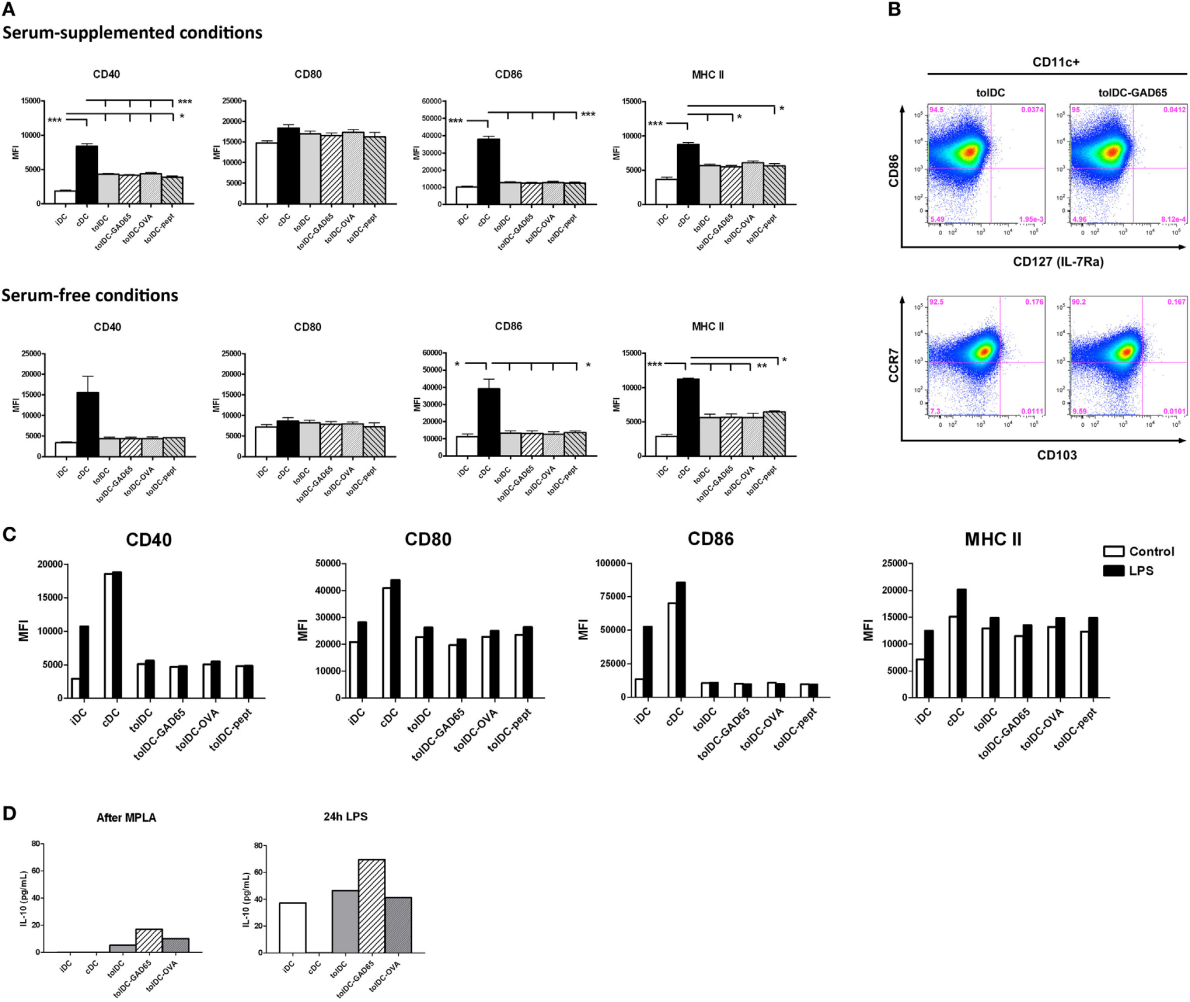
Phenotypic characteristics, anti-inflammatory cytokine profile, and stability of tolDCs and antigen-loaded tolDCs. **(A)** Expression of maturation markers CD40, CD80, CD86, and MHC II on immature bone marrow-derived dendritic cells (iDCs), control matured bone marrow-derived dendritic cells (cDCs), tolDCs, tolDCs-GAD65, tolDCs-OVA, and DCs loaded with 1 µg/mL of glutamic acid decarboxylase 65 (GAD65)-immunodominant peptide no. 35 (tolDCs-pept), cultured in serum-supplemented (RPMI-1640 with 10% fetal bovine serum) and SF conditions, was determined by surface staining of live cells and flow cytometry. Data are expressed as mean fluorescence intensities (MFI) from 4 to 5 (serum-supplemented conditions) or 2 to 3 (SF conditions) experiments ± SEM, *p < 0.05, **p < 0.01, ***p < 0.001. **(B)** Example dot plots from flow cytometry analyses of additional surface markers, i.e., CD103, CCR7, and IL-7Ra (CD127) on tolDCs and autoantigen-loaded tolDCs-GAD65. **(C)** Stability test of above listed types of DCs was carried out by additional 24 h culture (Control) or restimulation with 1 µg/mL lipopolysaccharide (LPS). Changes in expression of maturation markers CD40, CD80, CD86, and MHC II were assessed by flow cytometry and are displayed as MFI. **(D)** Interleukin 10 (IL-10) release after MPLA activation or 24 h LPS restimulation (stability test) by iDCs, cDCs, tolDCs, tolDCs-GAD65, and tolDCs-OVA. Data are expressed as means from two to four parallel cell cultures.

Expression of surface maturation markers on DCs such as costimulatory (CD80, CD86), activation (CD40) and antigen-presenting (MHC II) molecules was carried out by flow cytometry. As shown in Figure 5A, unloaded tolDCs as well as antigen-loaded tolDCs-GAD65, tolDCs-OVA, and tolDCs-pept exhibited similar pattern of these markers reflecting their immature to semi-matured phenotype in both serum-supplemented and SF conditions. The CD40 expression was substantially increased in cDCs compared to both iDCs and all groups of tolDCs in serum-supplemented conditions (*p* < 0.01, *p* < 0.001), whereas the differences were not significant in SF conditions due to a greater variability in cDCs values. There were no differences in CD80 expression, whereas CD86 was significantly upregulated on cDCs compared to iDCs and tolDCs in both serum-supplemented and SF conditions (*p* < 0.05, *p* < 0.001). A similar pattern was seen in expression of MHC II, where again cDCs exhibited statistically significantly increased levels compared to iDCs and all groups of tolDCs, irrespective of serum conditions (*p* < 0.05, *p* < 0.001, *p* < 0.001). Although not significant, of note is a slight increase of MHC II expression in all groups of tolDCs generated by our protocol compared to iDCs in both culture conditions. There were no differences between the serum-supplemented and SF cultures except a remarkably lower CD80 expression in all types of DCs cultured in SF conditions. The replacement of vitamin D2 by vitamin D3 in our protocol did not alter the pattern of maturation markers of tolDCs cultured in serum-suppl. conditions (Figure S1 in Supplementary Material). To conclude, antigen-unloaded tolDCs as well as antigen-loaded tolDCs-GAD65, tolDCs-OVA, and toldDCs-pept exhibited the same pattern of decreased maturation markers compared to cDCs in serum-supplemented and SF conditions.

We have examined expressions of other markers related to migration, mucosal homing, or induction of regulatory T-cell responses in antigen-loaded tolDCs-OVA and tolDCs-GAD65 and unloaded tolDCs. We found no substantial difference in expression of CCR5, CCR7, CD103, IL-7Rα, and c-kit among the tolDC-GAD65, tolDC-OVA and unloaded tolDC groups (some data shown in Figure 5B). None of the surface markers was differentially expressed among the three groups of tolDCs.

In order to determine whether tolDCs are resistant to additional maturation stimuli, the stability of unloaded tolDCs and antigen-loaded tolDCs was tested by 24-h restimulation with 1 µg/mL LPS, while 24 h-left unstimulated cells we used as controls. Figure 5C shows that irrespective of antigen loading, both tolDCs as well as tolDCs-GAD65, tolDCs-OVA and tolDCs-pept were refractory to LPS restimulation as documented by CD40 and even more CD86 expressions. Small increase in CD80 and MHC II expression could be noted after the prolonged LPS exposure; however, it was similar for all groups of tolDCs tested. While cDCs expressed already high levels of the examined maturation markers that could be only moderately further increased by LPS, iDCs showed substantial upregulation of CD40 and CD86 and to a lesser extent also CD80 and MHC II. Thus, all groups of tolDCs displayed a stable phenotype, irrespective of antigen loading.

We have also assessed secretion of IL-10 and IL-12 at the end of our DC-generation protocol, i.e., after final MPLA activation as well as after 24 h LPS restimulation (Figure 5D). Compared to cDCs both antigen-unloaded tolDCs as well as antigen-loaded tolDC-GAD65 and tolDC-OVA produced similar and distinct levels of IL-10 on day 8 after MPLA activation that were markedly increased by LPS restimulation (Figure 5C). No detectable IL-12 was found in all tolDC culture conditions, whereas cDCs produced 21.4 ± 1.1 pg/ml of IL-12 after MPLA activation (data not shown).

### Allogeneic Proliferative Responses and INF-γ Induction by tolDCs and Antigen-Loaded tolDCs

**Figure 6:**
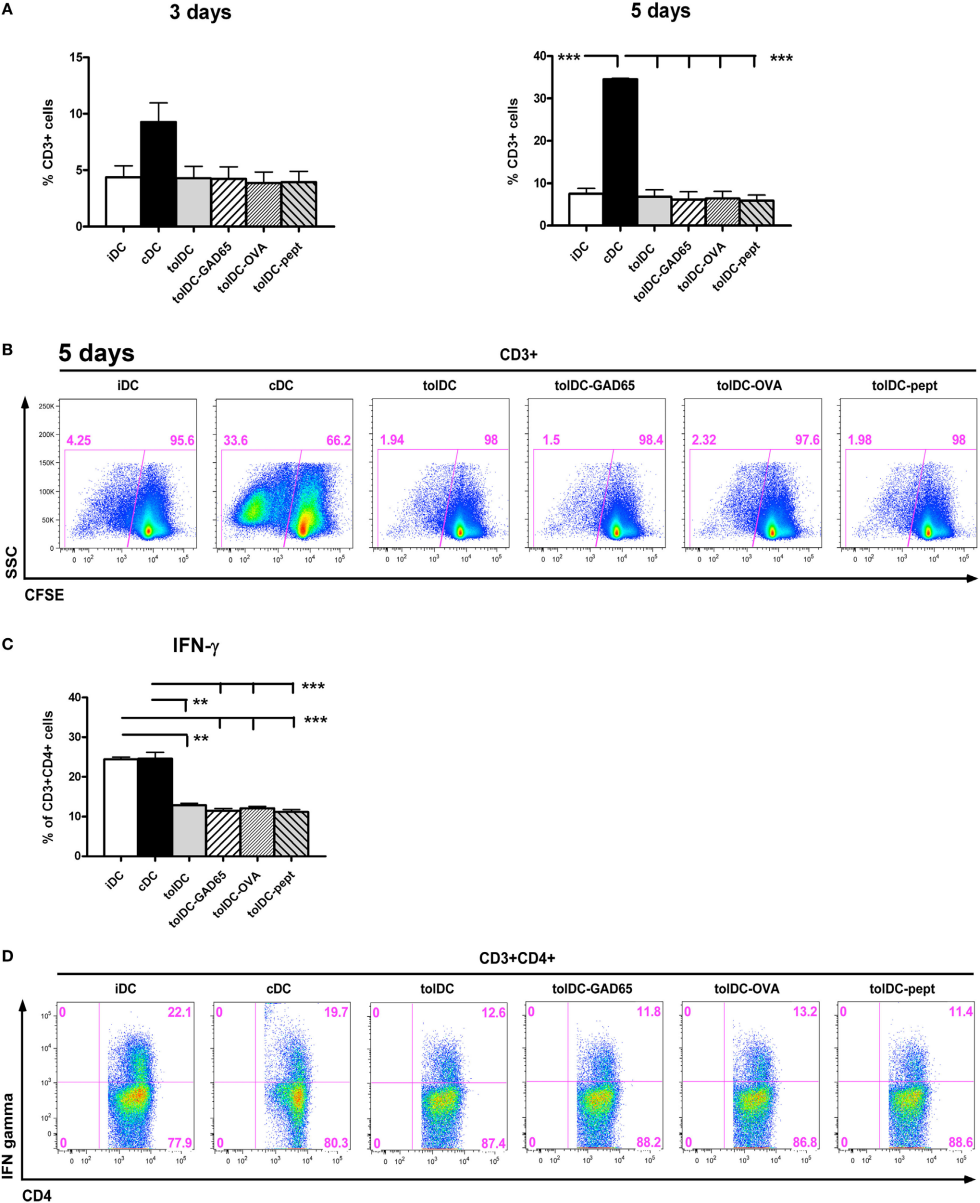
Allogeneic T cell proliferation and IFN-γ production by stimulation with tolDC vs. antigen-loaded tolDCs. **(A)** Allogeneic proliferative responses of immature bone marrow-derived dendritic cells (iDCs), control matured bone marrow-derived dendritic cells (cDCs), tolDCs, tolDCs-GAD65, tolDCs-OVA, and tolDC-pept were assessed by coculture of CFSE-labeled splenocytes (6- to 8-week-old C57BL/6 females) with DCs (8-week-old non-obese diabetes females) at 10:1 ratio for 3 and 5 days. Proliferation was measured as CFSE dilution in live CD3^+^ cells by flow cytometry. Splenocytes cultured alone were used as a control. All experiments were carried out in the serum-supplemented RPMI-1640 medium. Data are expressed as mean percentage of CFSElowCD3^+^ cells ± SEM of four experiments, **p < 0.01, ***p < 0.001. **(B)** Example of proliferation analysis by the flow cytometry of CFSE-labeled CD3^+^ splenocytes. **(C)** Induction of INF-γ in allogeneic CD4^+^CD3^+^ T cells was measured after 5 days of coculture with iDCs, cDCs, tolDCs, tolDCs-GAD65, tolDCs-OVA, and tolDCs-pept, following 4-h restimulation with phorbo-12-myristate-13-acetate/ionomycin by intracellular staining and flow cytometry analysis. Data are expressed as mean percentage of CD4^+^CD3^+^ cells ± SEM of four experiments, **p < 0.01, ***p < 0.001. **(D)** Example flow cytometry data of allogeneic induction of IFN-γ by DCs within CD3^+^CD4^+^ splenocytes.

The tolerogenic properties of tolDCs were evaluated by allogeneic proliferative T-cell responses. Splenocytes from C57BL/6 mice were cocultured with all groups of tested DCs in 10:1 ratio for 3 and 5 days and proliferation of CD3^+^ cells was assessed by flow cytometry. As shown in Figures 6A,B, the cDCs induced strong proliferative responses on day 3 and day 5 (9.28 ± 3.38 and 34.53 ± 0.51%, respectively). On the other hand, cocultures with iDCs and all groups of tolDCs—i.e., unloaded tolDCs, tolDCs-GAD65, tolDCs-OVA, and tolDCs-pept led to substantially reduced and comparable levels of proliferation of allogeneic splenic T cells. Thus, both iDCs and all groups of tolDCs induced statistically significantly lower proliferation of T cells on day 5 (*p* < 0.001). A similar pattern could be noted on day 3, but not statistically significant due to a greater variation of values in the cDC group (Figure 6A).

To further assess the tolerogenic properties of the tested DCs, we have determined induction of IFN-γ by CD4^+^ T cells in allogeneic settings. After 5 days, both cDCs and iDCs induced high proportion of IFN-γ producing CD4^+^ T cells (Figures 6C,D), whereas all groups of tolDCs were characterized by statistically significantly reduced percentages of IFN-γ-producing CD4^+^ T cells at level of *p* < 0.001, *p* < 0.001 for iDCs and *p* < 0.001 for cDCs (Figure 6C). In conclusion, all groups of tolDCs were characterized by a similar and statistically significant reduction of allogeneic proliferative T-cell responses and decreased IFN-γ induction.

### Dying Pattern of Antigen-Loaded tolDCs Compared to Unloaded tolDCs When Exposed to Autologous CD8^+^ or CD4^+^ Diabetogenic Splenocytes

In this experiment, we have tested the hypothesis that the opposite effect of tolDCs compared to antigen-loaded tolDCs-GAD65 or tolDCs-OVA on diabetes incidence may be due to an increased killing of antigen-loaded tolDCs by CD8^+^ or CD4^+^ T cells from within the cotransferred autologous diabetogenic splenocytes.

**Figure 7:**
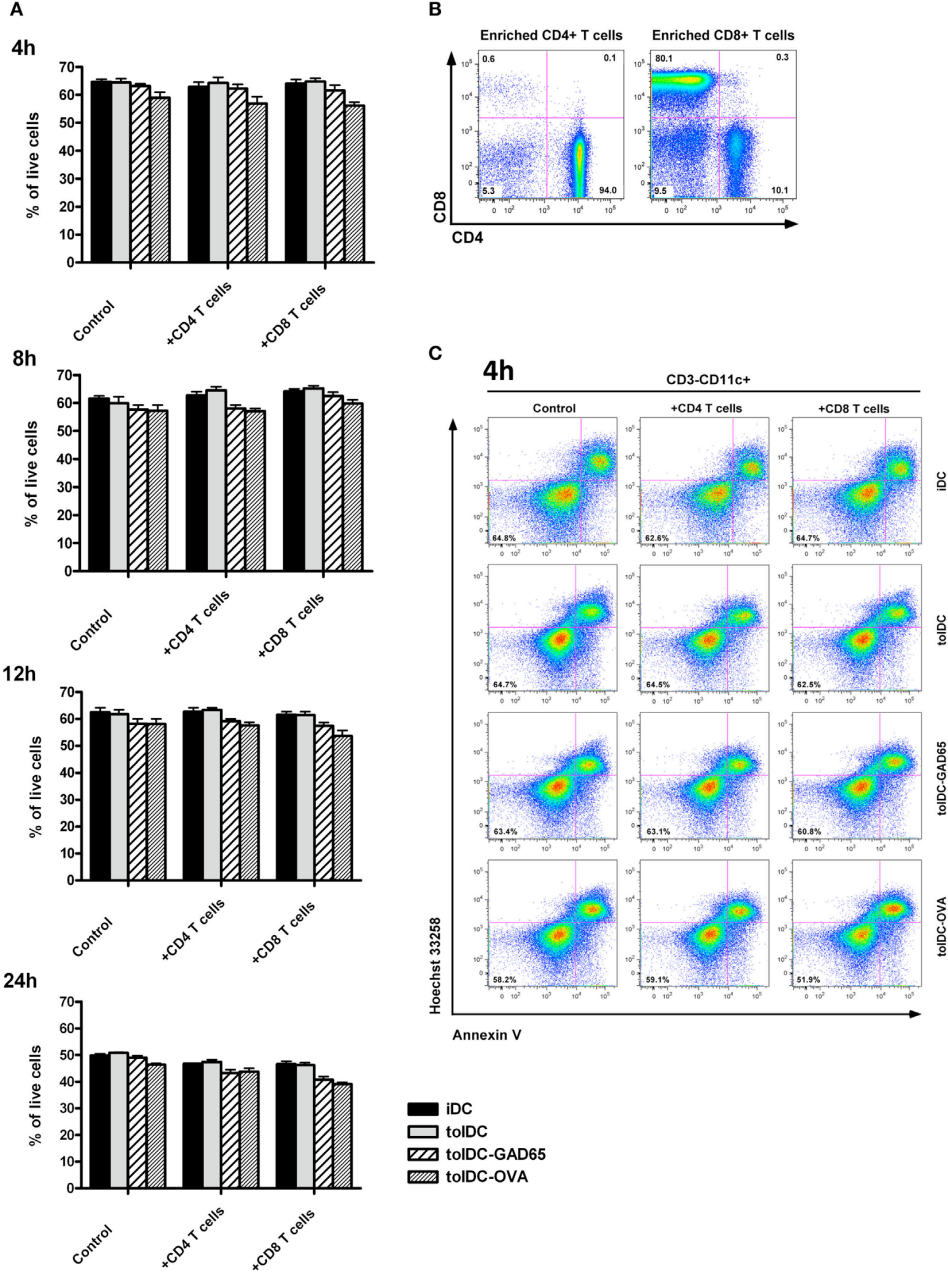
CD8^+^ and CD4^+^ T-cell-mediated *in vitro* killing of tolDCs vs. antigen-loaded tolDCs. **(A)** Equal number (5 × 10^6^) of immature bone marrow-derived dendritic cells (iDCs), tolDCs, GAD65-, or OVA-loaded tolDCs was mixed 1:1 with CD4^+^ or CD8^+^ splenic T cells (enriched by negative magnetic selection) and cocultured for 4, 8, 12, and 24 h. Dendritic cells cultured without splenic T cells were used as controls. Percentage of live nonapotopic cells was measured by flow cytometry as DCs (gated according to the FSC, SSC and CD3^−^CD11c^+^ parameters) stained double negative for Hoechst33342^−^ and AnnexinV^−^. Data are expressed as mean ± SEM of two to three experiments, *p < 0.05, **p < 0.01, ***p < 0.001. **(B)** Example of CD4^+^ and CD8^+^ T cell enrichment by negative magnetic selection. **(C)** Example of flow cytometry analyses of CD3^−^CD11c^+^AnnexinV^−^Hoechst33342^−^ cells at the 4 h timepoint.

Thus, iDC, tolDCs, tolDCs-GAD65, and tolDCs-OVA were cocultured with autologous CD8^+^ or CD4^+^ splenic T cells for 4, 8, 12, and 24 h. The purity of enriched CD4^+^ and CD8^+^ T cells by negative magnetic selection in this experiment was 94 and 80%, respectively (Figure 7B). The flow cytometry analysis of iDCs, tolDCs, and tolDCs-GAD65 or tolDCs-OVA documented relatively small changes in proportions of live non-apoptotic DCs (CD3^-^CD11c^+^AnnexinV^−^Hoechst33342^−^) among controls cultivated for additional 4, 8, 12, and 24 h (Figures 7A,C). There were also no statistically significant differences in percentage of live non-apoptotic DCs among the 4, 8, 12, and 24 h time points in general (Figure 7A). Compared to unloaded tolDCs, cocultures with CD8^+^ T cells led to slightly lowered percentage of live non-apoptotic tolDCs-GAD65 4 (3.23%), 8 (2.7%), 12 (4.0%), and also 24 h (5.5%), whereas a more substantial decrease was observed in the tolDC-OVA group after 4 (8.6%), 8 (5.3%), 12 (7.8%), and also 24 h (7.1%) (Figures 7A,C). Lower percentages of live non-apoptotic antigen-loaded tolDCs-GAD65 and tolDCs-OVA were also present after cocultures with CD4^+^ T cells (Figure 7A). Although the differences were not statistically significant, these percentages may reflect some CD8- and CD4-mediated killing of tolDCs-OVA or tolDCs-GAD65. However, similar but much less pronounced pattern of differences could also be seen in the control DCs cultured without autologous CD4^+^ or CD8^+^ T cells (Figures 7A,C). In another experiment, differences in percentage of live non-apoptotic antigen-unloaded tolDCs and antigen-loaded tolDCs-GAD65 and tolDCs-OVA after 4, 8, 12, and 24 h were also assessed in cocultures with whole autologous splenocytes at the ratio 3:5, to better reflect settings used for i.p. administrations to NOD-SCID mice. Similarly to data shown in Figure 7A, slightly lower percentage of antigen-loaded tolDCs-GAD65 and tolDCs-OVA compared to unloaded tolDCs were detected (Figure S2 in Supplementary Material), suggesting that some killing of antigen-loaded tolDCs may occur.

### Migration of Antigen-Loaded tolDCs-GAD65 and tolDCs after i.p. Administration in NOD Mice

**Figure 8:**
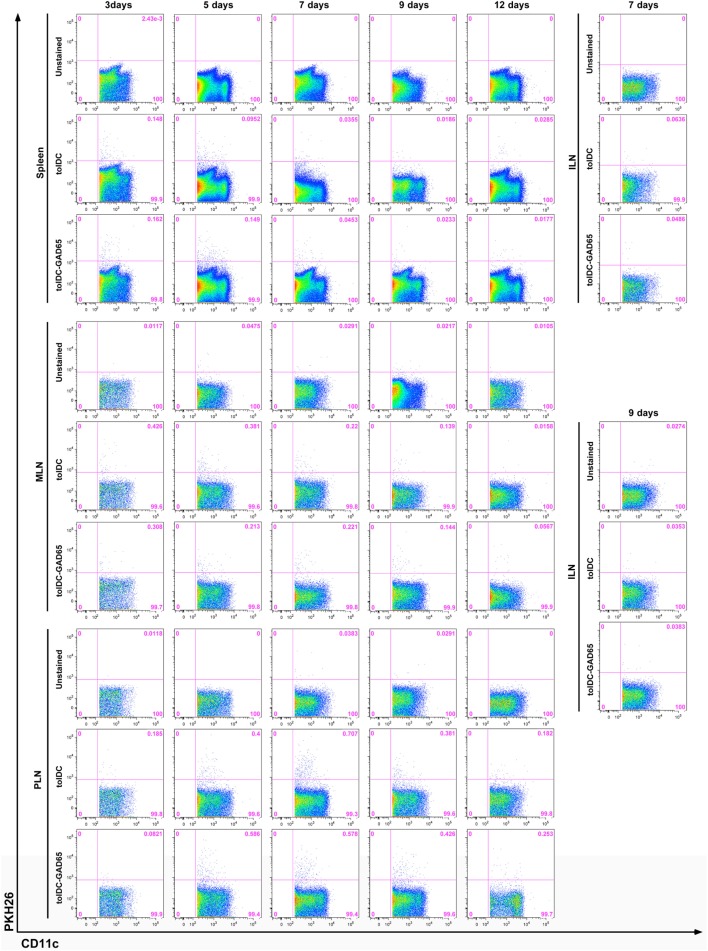
*In vivo* migration of PKH26-labeled tolDCs and tolDCs-GAD65. Bone marrow-derived dendritic cells were prepared from 8-week-old non-obese diabetes (NOD) mice. Tolerogenic DCs and tolDCs-GAD65 (1 µg/mL) were labeled with fluorescent PKH26 dye and 5 × 10^6^ cells were applied i.p. (left side of the belly) to 6-week-old NOD females. Unlabeled tolDCs were used as a negative control. FACS detection of PKH26^+^ cells was carried out on cell suspensions from spleen, mesenteric lymph nodes (MLNs), pancreatic lymph nodes (PLNs), and systemic inguinal lymph nodes (ILNs) after 3, 5, 7, 9, and 12 days (three mice per group for day 12, five mice per group for all other timepoints). Following doublets exclusion cells were gated according to the FSC-A and SSC-A parameters and dead cells were excluded by Hoechst 33258. PKH26^+^ tolDCs and tolDCs-GAD65 are displayed as percentage of live CD11c^+^ cells (1–2 × 10^6^ events per sample). Example of a larger (no. of time points) of two independent experiments.

The difference in the diabetes-preventive properties of unloaded tolDCs compared to antigen-loaded, e.g., tolDCs-GAD65 and tolDCs-OVA might be due to an altered migration pattern and/or trafficking dynamics of these cells after i.p. application. To test this hypothesis, we have investigated migration pattern of tolDCs in NOD mice. Tolerogenic DCs were prepared from bone marrows of NOD mice, then tolDCs and antigen-loaded tolDCs-GAD65 were labeled with the PKH26 dye for *in vivo* tracking and injected i.p. at dose of 5 × 10^6^ to 6-week-old NOD mice. Not surprisingly, the flow cytometry analysis revealed labeled PKH26^+^CD11c^+^ cells first (on day 3) in the spleen of NOD mice injected either with tolDCs or tolDCs-GAD65; 0.148 and 0.162%, respectively (Figure 8). While the number of the few PKH26^+^CD11c^+^ cells within spleen declined on day 7 in both tolDC and tolDC-GAD65 injected animals, there was a prolonged accumulation of the PKH26^+^ tolDC and tolDC-GAD65 cells in mucosal, MLNs from day 3 (0.426 and 0.308%), and 5 (0.381 and 0.213), to day 7 (0.220 and 0.221), while declining on day 9 (Figure 8). A similar pattern of trafficking to spleen and MLNs, but with a peak of accumulation of PKH26^+^CD11c^+^ cells in MLNs on day 5 was observed in a second (smaller scale) experiment (data not shown).

In both experiments, the highest percentage of PKH26^+^CD11c^+^ cells was found in the pancreas draining PLNs, irrespective of GAD65 antigen loading. As shown in Figure 8, the proportion of PKH26^+^ tolDCs and tolDCs-GAD65 in PLNs was 0.400 and 0.586 on day 5, 0.707 and 0.578 on day 7, and 0.381 and 0.426 on day 9, respectively. Only very few PKH26^+^CD11c^+^ positive cells were detected in PLNs on day 12, perhaps also due to the DC survival time *in vivo*. PKH26^+^CD11c^+^ cells were practically absent in the control ILNs (only data from day 7 and 9 are shown in Figure 8).

In conclusion, we have not found any differences in the migration pattern of diabetes-preventive tolDCs compared to tolDCs-GAD65, that are not effective in the disease prevention in the NOD-SCID model of adoptive transfer of diabetes. Both tolDCs and tolDCs-GAD65 appeared first in the spleen (day 3), stayed longer in MLNs and from day 5 accumulated most in the pancreas draining PLNs, where they stayed for up to 9 days, with the highest accumulation of the PKH26^+^CD11c^+^ cells on days 5 and 7. Both types of these tolDCs equally trafficked within the mucosal lymphoid compartment, preferentially to PLNs, and were not found in ILNs.

## Discussion

In this study, we have shown that antigen-unloaded tolDCs consistently decreased diabetes transfer in the NOD-SCID model of adoptive cotransfer of diabetes (Figures 1–3). On the other hand, tolDCs loaded with GAD65, with GAD65-immunodominant peptide no. 35, but also with a control protein OVA, all decreased their diabetes-preventive properties. Regardless their functional differences in the diabetes cotransfer model of NOD-SCID mice, the unloaded tolDCs and all three groups of antigen-loaded tolDCs displayed tolerogenic phenotype with IL-10 secretion and very similar effects in alloreactive T cell assays as well as remained stable after restimulation with LPS (Figures 5 and 6).

Unlike in animal models of EAE (22, 28) as well as in the models of experimental arthritis (24, 29), in which most of the tolDC-treatments are carried out in an autoantigen-specific manner, this is different in the field of T1D. Several studies documented diabetes prevention by antigen-nonspecific tolDCs (10, 15, 30, 31). While Tai et al. showed that tolDCs (prepared without final activation) in the presence of GM-CSF in combination with IL-10, but not IL-4, prevented diabetes in NOD mice (12), two other studies documented that antigen-nonspecific tolDCs prepared in GM-CSF + IL-4 (without final activation) prevented diabetes in NOD recipients (8, 9). Our diabetes-preventive unloaded tolDCs were prepared in the presence of GM-CSF + IL-4, further treated with dexamethasone and vitamin D2 and stabilized by a final activation with MPLA. On the other hand, not so many studies addressed T1D prevention by antigen-specific tolDCs. Tolerogenic DCs loaded with apoptotic bodies from the NIT-1 beta-cell line were effectively used in diabetes prevention in the RIP-IFN-β transgenic NOD mice (13); however, by this approach tolDCs are not exposed to a single antigenic entity. Lo et al. reported peptide-pulsed tolDCs preventing diabetes in NOD mice by using a peptide from an ignored GAD65 sequence and nonstabilized immature DCs (32). Peptide-specific approach was nicely documented in the humanized transgenic HLA-DR4 mouse model, in which i.d. application of GM-CSF + IL-4 + Vitamin D3-generated, LPS-activated tolDCs loaded with the proinsulin peptide C19-A3 reversed the break of tolerance as documented by decreased proliferation and peptide-specific induction of IL-10 (33). Finally, i.p. application of GM-CSF + IL-10-generated (without MPLA activation) tolDCs loaded with immunodominant insulin B chain peptides prevented diabetes in NOD mice by *in situ* induction of Foxp3^+^ Tregs, when cultured in autologous mouse serum (11).

This study as well as their previous paper (26) addressing the effect of nonautologous (FBS) vs. autologous sera on tolDC mechanisms of action (Th2 shift vs. induction of Tregs) together with data by Feili-Hariri et al. (9), who reported no additional beneficial effect of antigen-specific tolDCs compared to unloaded tolDCs (both cultured in FBS medium) pointed toward a nonspecific shift to Th2 immune responses probably due to presentation of FBS-related antigens. Thus, we tested our tolDC protocol also in SF conditions by using the CellGro medium, which is often used for DC cultures in human trails. As shown in Figure 3, we found no significant differences in SF settings, i.e., all antigen-loaded tolDCs (tolDCs-GAD65, tolDCs-OVA, tolDCs-pept) were again ineffective, whereas unloaded tolDCs led to a reduction of diabetes. Tolerogenic DCs cultured in SF conditions displayed slightly better diabetes-preventive effect (Figure 3A).

Several protocols generating stable tolerogenic DCs have been reported in the literature. These include, e.g., longer protocols based on the use of vitamin D3 such as an 8-day protocol with a continuous presence of vitamin D3 (34), a 10-day protocol with vitamin D3 and dexamethasone added only during last 16 h of LPS activation (24), vitamin D3 added from day 0 a dexamethasone on day 1 (35) or shorter one with dexamethasone added on days 3 and 6 and vitamin D3 on day 6 during final maturation (36), or a protocol using dexamethasone alone (37). In this study, we have used a protocol of tolDCs prepared in the presence of both GM-CSF and IL-4, added vitamin D2/dexamethasone and stabilized by final activation with MPLA. Our previous papers from overlapping groups of authors documented that human tolDCs prepared by this protocol (GM-CSF + IL-4 with added vitamin D2/dexamethasone and MPLA activation, in SF medium) led to induction of stable antigen-specific (GAD65) T cell hyporesponsiveness as well as induction of suppressive Tregs. Unloaded tolDCs prepared by this protocol prevented diabetes in the NOD-SCID model (38). This protocol also suppressed proliferation and induced IL-10 producing Tregs in a human allogeneic system. Good stability of these tolDCs in inflammatory environment was controlled by multiple signaling pathways including p38 MAPK, ERK1/2, mTOR, STAT3 and mTOR-dependent glycolysis (20). In the study by Sochorová et al. (39) vitamin D2 or vitamin D3 was added during LPS-induced activation of tolDCs. These tolDCs exhibited similar tolerogenic properties compared to tolDCs generated in the presence of vitamin D3 or vitamin D2 from the beginning of the cultivation (see also Figure S1 in Supplementary Material).

As shown in Figure 5, all groups of the NOD tolDCs were stable after 24 h restimulation with 1 µg/mL of LPS, secreted IL-10 (especially after restimulation with LPS) and displayed an immature phenotype in both serum-supplemented and SF conditions. In both culture conditions, tolDCs as well as all groups of antigen-loaded tolDCs displayed slightly increased expression of MHC II than iDCs. This increase of MHC II expression is not disadvantageous if tolDCs are stable. A similar pattern in MHC II expression was reported in dexamethasone-induced human tolDCs (40) or dexamethasone/vitamin D3 mouse tolDCs in experimental arthritis (24). The only notable difference between the tolDCs cultured in serum-supplemented and SF conditions was substantially decreased expression of CD80 in SF (Figure 5A). A comparative study on human clinical grade tolDCs (activated by a cytokine mix) showed that both dexamethasone and vitamin D3 produced stable tolDCs that suppressed allogeneic proliferation and IFN-γ induction by T cells (40). Similarly, García-Gonzales et al. (37) presented data on dexamethasone and MPLA activated stable human tolDCs with reduced allogeneic proliferation and IFN-γ induction (a 5-day protocol, dexamethasone added for last 48 h, without a vitamin D, 24 h MPLA activation). Our tolDCs, irrespective of antigen loading, displayed comparable parameters (Figures 5A,C,D and 6).

Several studies documented that DC cultivation with GM-CSF and IL-4 is favorable over GM-CSF alone, resulting in better tolerogenic properties and a more mature phenotype of tolDCs (9, 41, 42). The importance of IL-4 for induction of an increased stimulatory potential of DCs was documented by Wells et al. (43). GM-CSF + IL-4 generated DCs transduced with IL-4 were able to prevent diabetes in NOD mice with advanced insulitis (44). Gene array analyses revealed several differences including increased expression of costimulatory molecules, CD200, Ym-1 (marker of alternative macrophage activation), and different pattern of cytokine and chemokine expression by GM-CSF + IL-4 DCs (45). NOD tolDCs generated by GM-CSF + IL-4 and vitamin D3 were shown to induce Foxp3^+^ Tregs and IL-10 expression *in vitro* (34).

Our observation that antigen-loaded tolDCs are not so effective is not a complete surprise among studies dealing with tolDC therapy in animal models of T1D. There are scattered reports of antigen-loaded tolDCs being ineffective in the disease prevention, however, these findings were not much discussed or followed up. In 1999, Feilli-Hariri M et al. showed that tolDCs prepared in GM-CSF alone were less effective than tolDCs cultured in GM-CSF + IL-4, however in both cultures tolDCs pulsed with a mixture of 3 peptides (2 of GAD65 and 1 from hsp60 sequences) were less effective than unloaded control tolDCs (9). Later, Machen et al. reported that tolDCs prepared in GM-CSF + IL-4 and by antisense oligonucleotides against DC’s surface costimulatory molecules reduced diabetes incidence in NOD mice, but not if they were coadministered with a lysate from the NIT-1 β cell line (8). *In vivo* stimulation of DCs by PEGylated TLR7 ligand (1Z1) delayed and reduced diabetes as well as insulitis when they were transferred to prediabetic NOD mice. However, in the Figure S2 in Supplementary Material, the authors also show that 1Z1 treated DCs pulsed with GAD65 peptide 515–524 significantly increased insulitis in NOD mice (9 weeks after transfer) compared to both control animals with no transfer of cells but also compared to mice treated with 1Z1 DCs only (10). Finally, in the study addressing the effect of FBS on mechanisms of tolDC-action in NOD mice, only GM-CSF and IL-10 generated tolDCs pulsed with 2 insulin B chain peptides prevented diabetes in NOD mice. When splenocytes from these protected animals were cotransferred with diabetogenic splenocytes to NOD-SCID recipients, they caused a more rapid 100% diabetes onset compared to controls receiving only diabetogenic splenocytes (11). Indeed the NOD-SCID model of adoptive cotransfer of diabetes and the spontaneous NOD mouse model differ, e.g., the presence of self T and B cells or possibly a lower proportion of transferred Tregs within the diabetogenic splenocytes that may alter the effect of tolDCs in the NOD-SCID model. Another mechanism to consider is a homeostatic expansion of transferred diabetogenic lymphocytes in immunodeficient settings. On the other hand, Machen et al. (8) documented that NOD mice injected first with NOD T cells followed by administration of tolDCs or control DCs displayed a significant increase in the number of total splenic CD4^+^CD25^+^ and CD25^+^CD62L^+^ regulatory cells only in the group injected with tolDCs. This finding together with the absence of differences in the prevalence and numbers of single CD4^+^ or single CD8^+^ cells between NOD-SCID groups treated with tolDCs and control DCs argues against homeostatic expansion as the basis of the increased prevalence of the CD4^+^CD25^+^CD62L^+^ cells. Since many studies were performed in the spontaneous NOD mouse model of T1D, we also tested unloaded and antigen-loaded tolDCs in NOD mice. Similar diabetes-preventive effect of antigen-unloaded but not antigen-loaded tolDCs (not only GAD65- but also OVA-loaded tolDCs) on diabetes prevention was documented (Figure 4). The above listed (8–11) scattered evidence about less effective or ineffective antigen-loaded tolDCs in the literature is also derived from the NOD mouse model.

We also addressed the possibility of bystander antigen presentation by APCs present within the diabetogenic splenocytes and cotransferred with antigen-loaded tolDCs to NOD-SCID recipients. Yet, by using purified diabetogenic T cells instead of whole splenocytes, we only observed a tendency for a bit more effective diabetes prevention by unloaded tolDCs (Figure 2). Another mechanism to consider was killing of antigen-loaded tolDCs by autologous CD8^+^ or CD4^+^ T cells present within the diabetogenic splenocytes. Although not statistically significant, decreased percentage of live non-apoptotic OVA- and to a lesser extent GAD65-loaded tolDCs compared to unloaded tolDCs was detected in cocultures with autologous CD8^+^ and CD4^+^ T cells (Figure 7). However, there is a possibility that antigens from dying antigen-loaded donor tolDCs are presented *in vivo* by recipient APCs (DCs) in an immunogenic fashion as reported by Smyth et al. (46), even in autologous settings. In NOD mice, these mechanisms could be further enhanced by a defect in tolerance induction by CD8^+^DCs that express higher levels of CD40 (47).

Our next experiment showed preferential mucosal migration of both unloaded tolDCs as well as antigen-loaded tolDCs-GAD65 and tolDCs-OVA with highest accumulation in PLNs (Figure 8). PLNs were shown to play a critical role in priming beta-cell-specific immune responses as, e.g., removal of PLNs prevented diabetes development in NOD mice (48), T cells from BDC2.5 T cell receptor transgenic mice, that are specific for a natural beta-cell antigen, proliferate exclusively in PLNs before onset of insulitis (49) and increased migration of mature tolDCs generated by vitamin D3 to PLNs of NOD mice was reported (34). NOD’s PLNs were also described to harbor increased number of merocytic mcDCs that induce T-cell activation and break T-cell tolerance to beta-cell antigens (50). Although we found no differences in surface expression of DC markers related to migration and mucosal homing between the unloaded tolDCs and antigen-loaded tolDCs-OVA or tolDCs-GAD65, of note is that our tolerogenic protocol as well as protocol based on dexamethasone alone followed by MPLA activation (37) led to increased CCR7 expression (Figure 5B). It has been reported that CCR7 (51) but not CD103 (52) is critical for mucosal (MLNs) homing of DCs.

Turner et al. have published interesting data documenting the importance of antigen dose on induction of Foxp3^+^ Tregs (low dose, 0.4 µM) or Foxp3^−^CD4^+^ T cells (high dose, 40 µM) in relation to weak and strong activation of Akt/mTor TCR signaling pathway. This effect was modulated by IL-6 and was present not only in GM-CSF + IL-4 tolDCs, but also in immature DCs cultured in GM-CSF only (53). The effect of a dosage might explain the decreased diabetes-preventive properties not only of GAD65- and pept-loaded tolDCs but also of tolDCs loaded with the control protein OVA (Figures 2 and 3A). These findings may correspond with a pattern of diabetes incidence of GAD65-loaded tolDCs presented in Figures 1 and 2. While the higher dose of 2 µg/mL of GAD65 led to 100% diabetes transfer and a more rapid onset of diabetes (Figure 1), tolDCs loaded with the lower dose of 1 µg/mL (Figure 2) precipitated lower (seven of eight mice) and slower transfer of diabetes than Controls. The antigen dose of 1–2 µg/mL in our experiments is, however, lower than in other studies using antigen-loaded tolDCs for T1D prevention that ranged from 10 µg/mL (11) to 3 × 60 μg/mL (9) or 10 µM (32). In animal models of EAE and rheumatoid arthritis (RA) doses of peptides ranging from 5 to 50 µg/mL and 1 to 50 µg/mL, respectively, were used for pulsing tolDCs (42). Nevertheless, controlling the outcome of a tolDC therapy by antigen doses alone would be a difficult task. Less protective effect of antigen-loaded tolDCs could be also due to a combination of factors, e.g., the dose, killing by autologous CD8^+^ and CD4^+^ T cells or presentation of antigens from dying antigen-loaded tolDCs by recipient APCs (46) and/or by homeostatic expansion of diabetogenic lymphocytes in the NOD-SCID model.

Although antigen-specific tolDCs are widely used in animal models of autoimmune diseases such as EAE or RA (42), it should be noted that T1D may differ in some aspects from other autoimmune diseases. Development of T1D seems to be more related to impaired, “missing” regulatory immune responses in genetically predisposed individuals (54). Interestingly, T1D does not fulfill one important of the Rose-Witebsky’s criterions of autoimmune diseases—induction of the disease by immunization with an autoantigen (55).

In conclusion, while tolDCs represent a very promising strategy for prevention or even early cure of T1D, our data together with previously published scattered evidence suggest that antigen loading decreases the disease-protective effect of tolDCs in animal models of T1D. *In vivo* testing of tolDCs is important as multiple factors may influence their therapeutical effects. Further studies are needed to shed more light on the mechanisms of antigen-specific tolDCs in animal models of T1D.

## Ethics Statement

This study was carried out in accordance with the recommendations of our institutional animal ethics office (Laboratory Animal Care and Use Committee of the Institute of Microbiology v.v.i., Academy of Sciences of the Czech Republic, approval ID: 94/2006 and 244/2009) in strict accordance with the Federation of European Laboratory Animal Science Associations guidelines.

## Author Contributions

All authors (DF, JG, TH, HK, RŠ, LP-J) contributed to the study concept, data acquisition and drafting the work. DF wrote the paper. DF, LP-J, and JG performed data analysis and interpretation of the data. LP-J and RŠ contributed to the critical revision of the manuscript. DF and LP-J are the guarantors of this work.

## Conflict of Interest Statement

LP-J, and RŠ are named inventors in a related patent, “Tolerogenic Dendritic Cells, Methods of Producing the Same, and Uses Thereof” PCT/EP2015/074536 which describes methods for the preparation of stable semi-mature tolerogenic DC. The other authors have no financial conflicts of interest.

## Abbreviations

cDC, control matured bone marrow-derived dendritic cell; iDC, immature bone marrow-derived dendritic cell; ILN, systemic inguinal lymph node; MLN, mesenteric lymph node; pept, GAD65-immunodominant peptide no. 35; PLN, pancreatic lymph node; tolDC, tolerogenic dendritic cell.
