# Supplementary figures and images for: Antigen Loading (e.g., Glutamic Acid Decarboxylase 65) of Tolerogenic DCs (tolDCs) Reduces Their Capacity to Prevent Diabetes in the Non-Obese Diabetes (NOD)-Severe Combined Immunodeficiency Model of Adoptive Cotransfer of Diabetes As Well As in NOD Mice
Source: Front Immunol. 2018 Feb 16;9:290. doi: 10.3389/fimmu.2018.00290 (PMC5820308; doi:10.3389/fimmu.2018.00290)

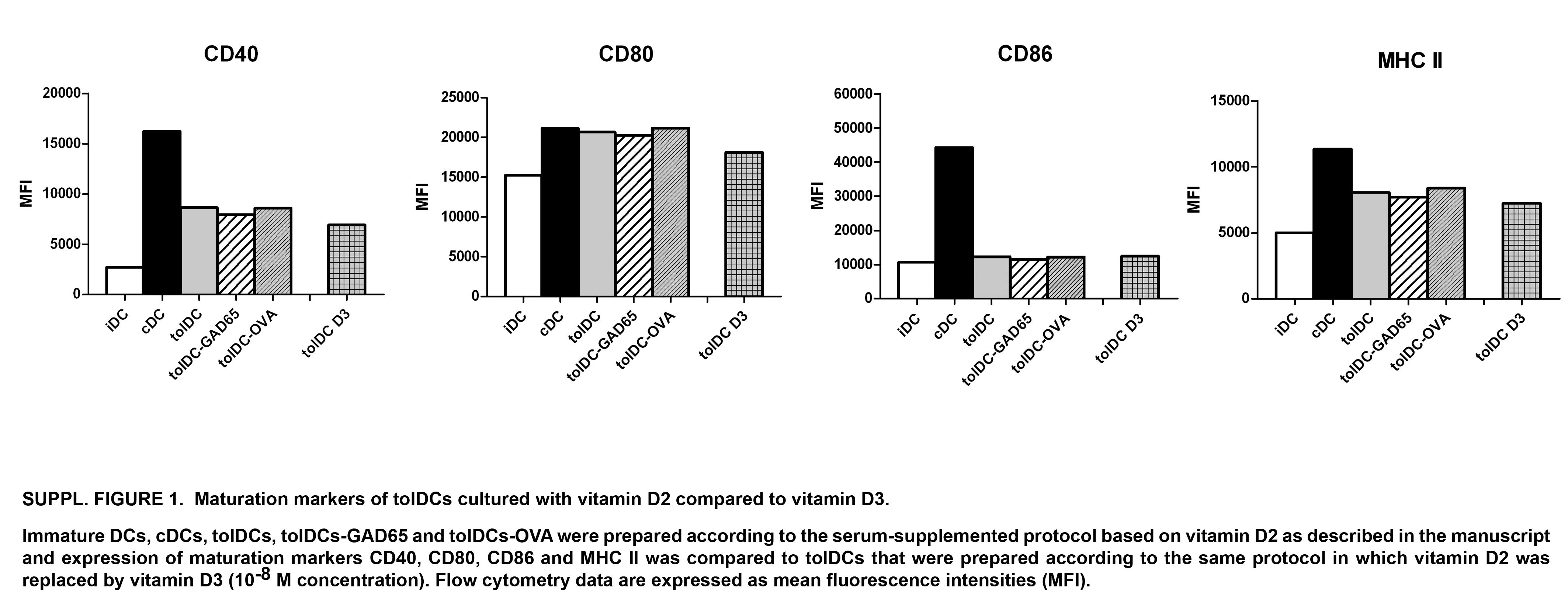

Supplement: Supplementary file 1 [file image_1.tif]

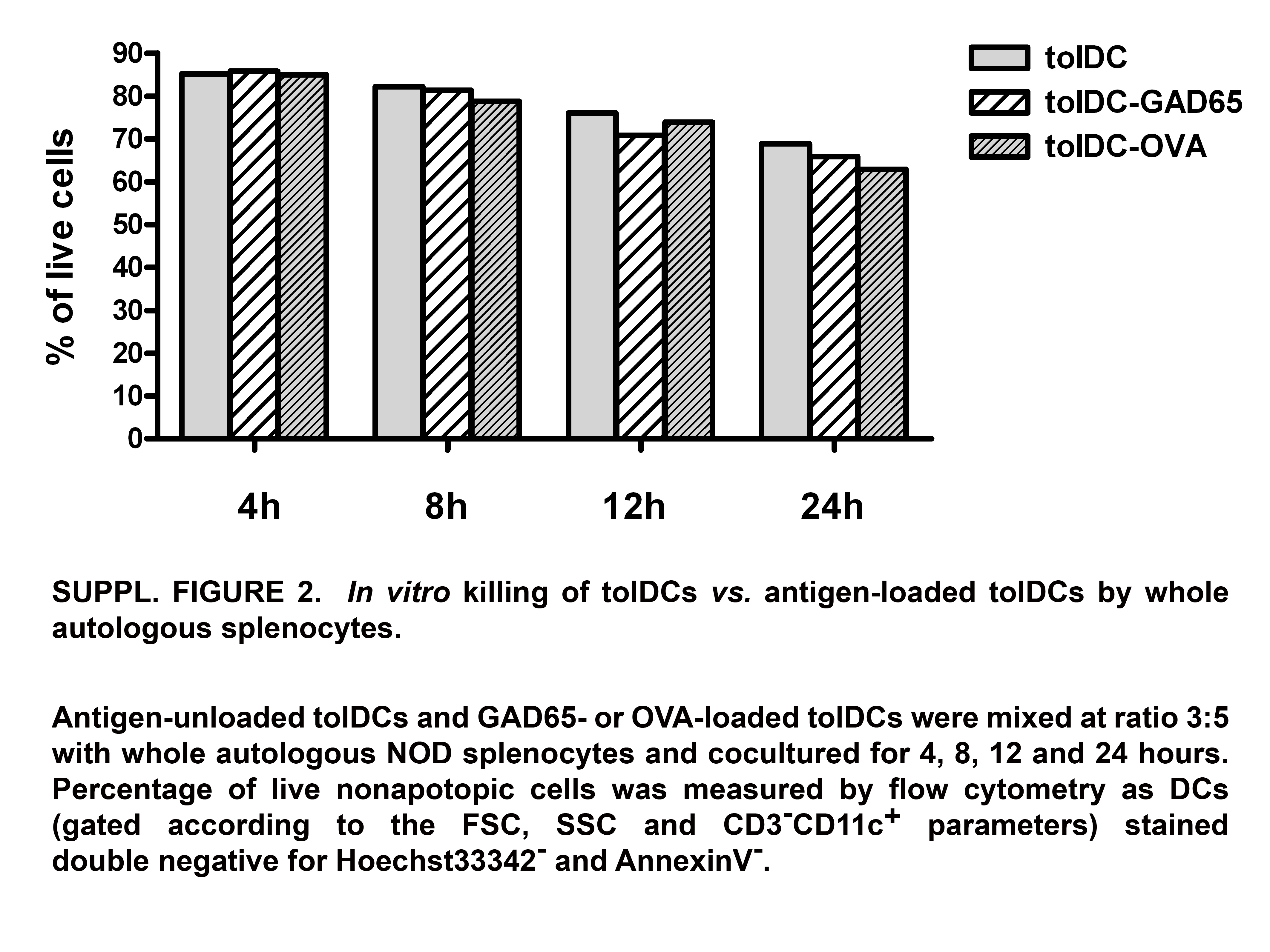

Supplement: Supplementary file 2 [file image_2.tif]
